# Multicell migration tracking within angiogenic networks by deep learning-based segmentation and augmented Bayesian filtering

**DOI:** 10.1117/1.JMI.5.2.024005

**Published:** 2018-06-13

**Authors:** Mengmeng Wang, Lee-Ling Sharon Ong, Justin Dauwels, H. Harry Asada

**Affiliations:** a Nanyang Technological University, Energy Research Institute, Singapore; b Singapore-MIT Alliance for Research and Technology, Singapore; c Nanyang Technological University, School of Electrical and Electronic Engineering, Singapore; d Massachusetts Institute of Technology, Department of Mechanical Engineering, Cambridge, Massachusetts, United States

**Keywords:** backward Kalman filters, coarse time-lapse phase-contrast images, convolutional neural networks, endothelial cell networks tracking, end-point confocal images, multiple hypothesis tracking

## Abstract

Cell migration is a key feature for living organisms. Image analysis tools are useful in studying cell migration in three-dimensional (3-D) *in vitro* environments. We consider angiogenic vessels formed in 3-D microfluidic devices (MFDs) and develop an image analysis system to extract cell behaviors from experimental phase-contrast microscopy image sequences. The proposed system initializes tracks with the end-point confocal nuclei coordinates. We apply convolutional neural networks to detect cell candidates and combine backward Kalman filtering with multiple hypothesis tracking to link the cell candidates at each time step. These hypotheses incorporate prior knowledge on vessel formation and cell proliferation rates. The association accuracy reaches 86.4% for the proposed algorithm, indicating that the proposed system is able to associate cells more accurately than existing approaches. Cell culture experiments in 3-D MFDs have shown considerable promise for improving biology research. The proposed system is expected to be a useful quantitative tool for potential microscopy problems of MFDs.

## 1 Introduction

Cell migration experiments can be performed in three-dimensional (3-D) microfluidic devices (MFDs).^1^ These devices, with channels allowing either fluid to flow or gel scaffolds to be injected, mimic the *in vivo* environments. In conventional two-dimensional (2-D) *in vitro* experiments, cells move freely and form a thin layer rather than a structure. In *in vivo* experiments, we can only see the formed structure. However, from the experiments in the 3-D MFDs, we can obtain the microscopy images of both the structure and the individual cells. As controlled reactions can be reproduced with a small volume of samples and reagents, MFDs are used for long-term studies of many biological processes. In this study, we consider cell migration during angiogenesis and develop an automated image analysis system to extract cell migration behaviors.

Angiogenesis is the formation of new blood vessels from pre-existing vessels.^2^ It is a critical process in growth, development, and cancer invasion. During angiogenesis, endothelial cells (ECs) lining an existing vessel (monolayer) will sprout out into the gel, in response to local chemical and mechanical stimuli.^3^^–^^5^ Our angiogenic experiments are conducted in MFDs, where the ECs have been shown to migrate in 3-D environments by Ong et al.^6^ Stained cells under fluorescent/confocal microscopy provide clear cell locations. However, staining inhibits cell proliferation and migration especially over long period.^7^ Therefore, we culture the unstained ECs and observe the angiogenic vessels by phase-contrast microscopy^8^ at 20× magnification daily over a period of 10 to 14 days.^9^ Each image has a resolution of 1532×2048  pixels. At the end point, we image the vessels by confocal microscopy after staining cell nuclei with Hoechst dye (Invitrogen) and actin with Alexa Fluor 488 Phalloidin (Invitrogen).

Automated image analysis tools to extract cell migration behaviors are useful for a wide range of biomedical research. Conventional image analysis systems focus on cell migration in 2-D *in vitro* environments, whereas our cells are cultured in MFDs. Unlike the cells in 2-D, our cells migrate within the 3-D collagen gel, remodel the gel, and form 3-D blood vessels. Moreover, these cells do not overtake each other due to the existence of tight cell junctions, and their migration is constrained by the vessels. This creates a unique problem that has never been addressed.

### 1.1 Existing Approaches

The existing automated image analysis techniques for cell segmentation and tracking can be classified into two categories: tracking by model evolution and tracking by detection. The former category detects and tracks the cells simultaneously by representing the cells with mathematical models, whereas the latter category first detects cell candidates in all the frames independently and then links the detected candidates into tracks.

In model evolution algorithms, the mathematically represented cell contours are smoothly evolved from one time frame to the next one, using a velocity term defined by the content of the “target frame.”^10^^–^^14^ In general, model evolution algorithms require a high imaging frequency to allow sufficient cell overlap between frames and accurate cell contour/boundary detection. These approaches are not applicable to our images due to our low imaging frequency (one day) and unclear cell boundaries (tight cell junctions).

**Fig. 1:**
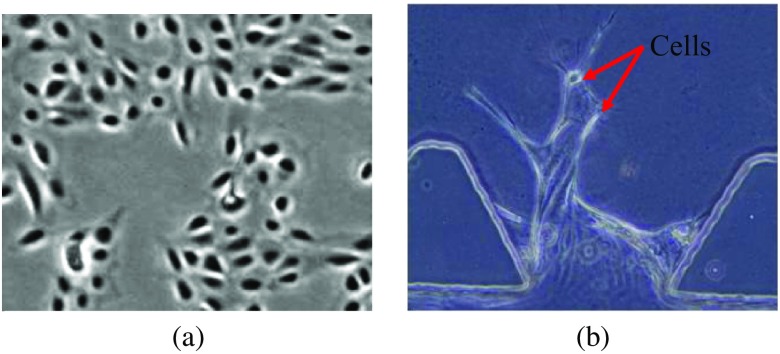
Examples of cells (a) on a 2-D surface and (b) within 3-D angiogenic vessels (red arrows point to cells).

In tracking by detection algorithms, cells are first detected in all the frames independently using thresholding of pixel intensities,^15^^,^^16^ edge detection,^17^ image restoration,^18^^–^^20^ or template matching.^21^ Kanade et al.^18^ applied image restoration to segment the cells on a 2-D flat surface from phase-contrast images, as shown in Fig. 1(a). Due to the different culture environments, the morphologies of our cells within angiogenic vessels are different [see Fig. 1(b)]. Moreover, the thickness of the MFDs is 120  μm, whereas the thickness of a cell on a flat surface is around 3 to 20  μm. Due to the artifacts of phase-contrast microscopy, imaging thicker specimens produces severe halo effects that reduce the visibility of our cell boundaries unlike the data by Kanade et al.^18^ In addition to that, some cells may be slightly out of focus. Therefore, the image restoration technique is not applicable for our images. A different approach is template matching; a template is compared to patches of a gray-level image by means of a match statistics evaluated for each patch.^22^ Euclidean distance, mean square difference, and support vector machine (SVM) can work as the template matching criteria for cell detection.^21^^,^^23^^–^^26^ Since we can easily obtain cell templates from end-point phase-contrast images by aligning the latter with their corresponding end-point confocal images, we apply convolutional neural networks (CNN) to detect cell candidates from the time-lapse phase-contrast images.

Subsequently, association algorithms, such as “nearest-neighborhood” association,^27^^,^^28^ mean-shift process,^29^^–^^31^ the Hungarian method,^32^ Viterbi algorithm,^33^ Kalman filtering,^34^ particle filtering,^35^ multiple hypothesis tracking (MHT),^18^^,^^36^^,^^37^ and active structured learning,^38^ can be used to determine the most likely cell correspondence between frames. These techniques are either applied to images of cells migrating on a 2-D surface or 3-D *in vivo* florescence images, which are far different from our 3-D *in vitro* application. In the works by Ong et al.,^6^ stained ECs and vessels were tracked using Bayesian filtering from time-lapse confocal images of 30-min intervals. However, our images are acquired once a day, which is much longer than most existing tracking systems. Therefore, tracking these cells is a challenge that has not been previously addressed.

### 1.2 Summary of Contribution

In this paper, we focus on tracking by detection algorithm to track the migrating ECs within angiogenic vessels formed in 3-D MFDs by linking the coarse time-lapse 2-D phase-contrast images and 3-D end-point confocal images. Since the ECs in 3-D MFDs can appear and disappear from our 2-D phase-contrast images, the main challenge is to accurately identify the cell candidates and track cell division and migration in 3-D (in focus/out of focus) over image sequences.

Our proposed system includes image registration, vessel segmentation, cell detection, and multiple hypothesis Kalman filtering, to track cell proliferation and migration within angiogenic vessels in different frames. As mentioned, we obtain time-lapse phase-contrast images and end-point confocal images of the angiogenic vessels from the experiments. The stained cells are easily recognized in the end-point confocal images. Therefore, instead of starting with the first time point like other cell tracking applications, we apply backward Kalman filter by initializing our tracks with the accurate cell locations obtained from the confocal images at the final time point where all proliferation has occurred. Furthermore, we incorporate biological knowledge to add constraints and to estimate the track probability during cell association:

• In our angiogenic vessels, cells cannot overtake the cells in front of them, whereas cells on a 2-D surface can move freely. This difference is one of the constraints during association.
• In conventional approaches, the increment of cells with time is only due to proliferation. However, for angiogenic experiments in 3-D MFDs, cells can migrate from cell–gel interface to form the angiogenic vessels. In other words, the increment of cells can be either due to proliferation or migration from cell–gel interface. Thus, we include an empirical equation in our tracking algorithm to estimate the probability that one cell is due to migration from cell–gel interface.

Since one phase-contrast image is recorded each day for each slot, biologists can conduct many experiments in parallel, where cell viabilities are well-maintained. Moreover, by backward tracking each cell in the 3-D network structure, cell lineage and trajectory information, linking the chemical and physical characteristics of a cell can be obtained in an automated manner.

The rest of this paper is structured as follows. In Sec. 2, we explain our proposed automated cell tracking system. In Sec. 3, we present numerical results on our experimental phase-contrast images. We offer concluding remarks in Sec. 4.

## 2 Automated Multicell Tracking System

**Fig. 2:**
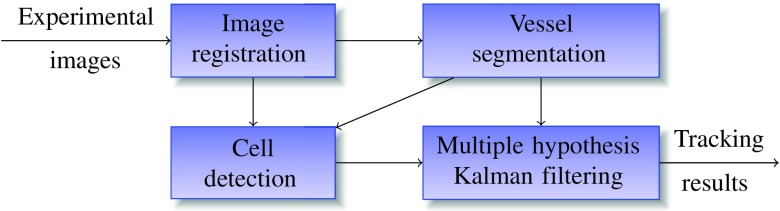
Diagram of the proposed automated tracking system: image registration to align all the experimental images, vessel segmentation to represent binary vessel shape, cell detection to label all cell candidates, and multiple hypothesis Kalman filtering to associate the detected cell candidates.

Our automated tracking system is shown in Fig. 2. First, we align and transform all the experimental images, including both phase-contrast images and the end-point confocal images, into one common coordinate system through image registration. We then apply morphological image processing algorithms to segment the binary vessel shape. This shape is converted to a medial axis transform (MAT) representation.^39^ Simultaneously, we train a CNN classifier to detect and label cell candidates in the phase-contrast images. Last, by combining backward Kalman filtering with MHT, we associate and track the detected cell candidates over sequences. From the tracking results, we can obtain cell lineage and trajectory information to understand cell characteristics better. In the following, we will discuss each component in detail.

### 2.1 Image Registration

**Fig. 3:**
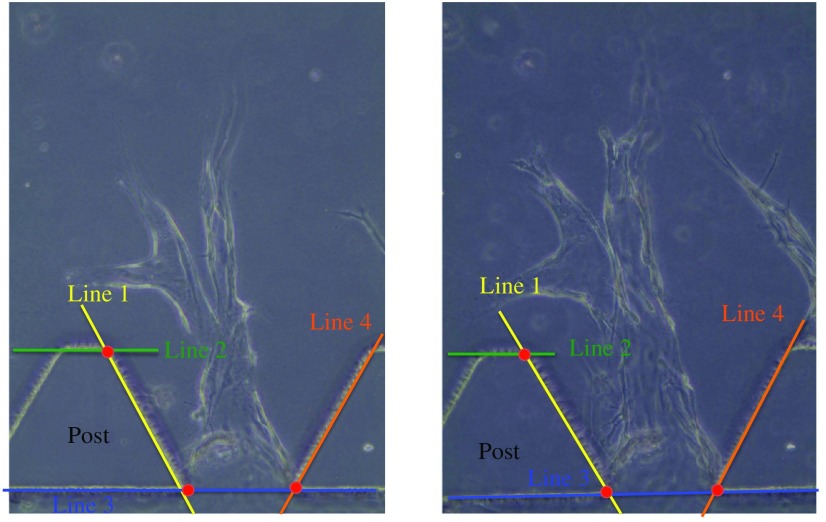
Experimental phase-contrast images of two successive frames. The trapezoidal post is designed to retain the gel within the microfluidic channel. Lines 1 to 4 can be detected and represented using Hough transform. The red points are the correspondence points used during image registration.

We acquired the experimental images manually at different days; hence, the angiogenic vessels are misaligned across the image sequences as shown in Fig. 3. We spatially register all the image sequences by transforming them into a common coordinate system, so that we can associate and track all the cells.

To register these images, we first extract salient features from all the images. We choose the intersections of the straight post edges (lines 1 to 4) as features, which are labeled in red in Fig. 3. To automatically detect these features, we apply the Hough transform to identify the straight lines representing the post edges. From these detected lines, the corresponding intersections between images are obtained. We then derive the scale, linear translation, and rotation parameters based on the intersections’ coordinates by iterative closest point to align all the images. The pixels below the identified post edges are marked to zero to avoid any post features to be detected as vessels or cells in the later stage.

### 2.2 Vessel Segmentation

**Fig. 4:**
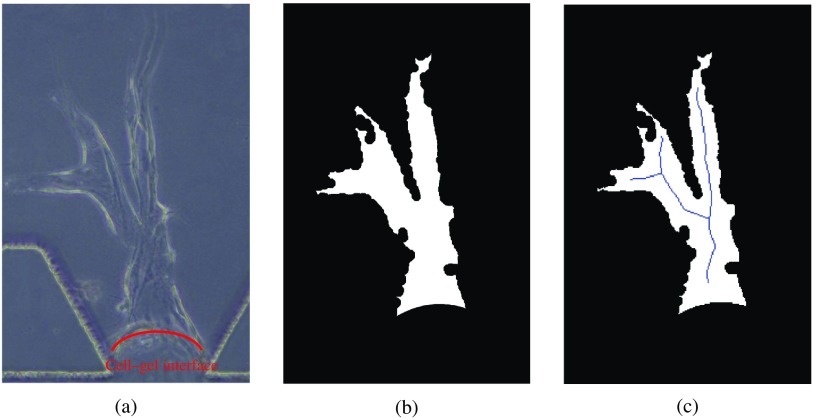
Examples of vessel segmentation: (a) cell–gel interface, (b) the segmented binary vessel shape, and (c) vessel shape with centerline.

We aim to investigate the behavior of cells within the gel. Hence, we do not need to segment cells under the cell–gel interface [see Fig. 4(a)]. Based on the observations, we fit a parabola to estimate the cell–gel interface in the experimental images. The parabola equation has the following form:

$$a_{1}{\left(x-a_{p}+a_{3}\right)}^{2}+a_{2},$$ (1)

where $a_{p}$ is the intersection point of lines 1 and 4 obtained during image registration, and $a_{1}$, $a_{2}$, and $a_{3}$ are the coefficients of the parabola. The best-fit is obtained by brute-force optimization.^1^ The pixels below the parabola are masked out as our region of interest is the area above the cell–gel interface.

Next, we convert the processed experimental images into binary images with the global image threshold computed using Otsu’s^40^ method. We obtain the segmented binary vessel shape [see Fig. 4(b)] through the following series of morphological operations in MATLAB™:^22^

• “imclose” to connect the gaps in the boundaries: structuring element is a 2-D disk-shaped structuring element with 15 pixels,
• “imfill” to fill the holes inside the vessel area, and
• “bwareaopen” to remove the noises that have fewer than P pixels: P=20,000.

These parameters are selected based on a set of training images, and moderate changes in these parameters do not lead to significant change in the binary vessel shapes.

We represent the segmented vessels shape with MAT by a centerline [see Fig. 4(c)] and radii.^39^ We can reconstruct the original vessel shape for visualization with the obtained MAT data set. Since the cells typically remain within the vessel, we can eliminate cell candidates outside the vessel during cell detection.

### 2.3 Cell Detection

**Fig. 5:**
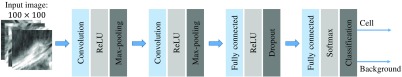
Architecture of the CNN classifier for cell detection.

CNN is a widely used deep learning approach in pattern-recognition and image-recognition problems.^41^ We construct a CNN architecture by stacking multiple layers (see Fig. 5) and train the CNN network to distinguish cell and noncell templates. The resulting CNN classifier is then used to detect the cell candidates from the time-lapse phase-contrast images.

**Fig. 6:**
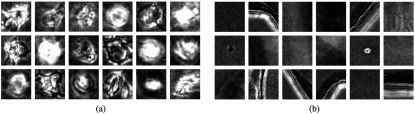
Examples of (a) cell and (b) noncell templates from phase-contrast images to train the CNN classifier. Size of each template is 100×100.

We use the end-point confocal images, where we can easily recognize the cells, to select our cell templates to ensure they are informative. By aligning the phase-contrast images with their corresponding confocal images, we can distinguish cell coordinates and noncell coordinates in the phase-contrast images. These coordinates are cropped as our templates, as shown in Fig. 6. After rotating the templates by 90 deg, 180 deg, and 270 deg, respectively, flipping them horizontally and vertically, and adding Gaussian noise, we obtain 3228 cell templates and 3372 noncell templates to train the CNN classifier. Each template has a size of 100×100, which is sufficiently large to fit each individual cell.

The architecture of a CNN varies depending on the types and numbers of layers included. Some common types of layers include convolution, ReLU, pooling, dropout, fully connected, softmax, and classification layer.

The convolution layers are the core building blocks of a CNN, which extract different features of the input.^41^^,^^42^ An image becomes a stack of filtered images after a convolution layer. ReLU is the abbreviation of rectified linear unit, which implements the function y=max(x,0) to set any negative input value to zero. It increases the nonlinear properties of the overall network without affecting the receptive fields of the convolution layer.^43^ The pooling layer serves to progressively reduce the spatial size of the representation, to reduce the number of parameters and amount of computation in the network and, hence, also to control overfitting. Max-pooling is the most common pooling operation, which partitions the input image into a set of nonoverlapping rectangles, and for each such subregion, outputs the maximum. After several convolution, ReLU, and max-pooling layers, the fully connected layer combines all of the features learned by the previous layers to classify the images.^44^ A dropout layer, which randomly sets input elements to zero with a given probability, follows the fully connected layer to reduce overfitting.^45^ The fully connected layer usually uses the softmax activation function (softmax layer) for a classification problem.^46^ The classification layer is the final layer, which uses the probabilities returned by the softmax activation function for each input to assign it to one of the classes.

The types and number of layers included in a CNN classifier depend on the training data. To get the most suitable architecture of our CNN classifier, we vary not only the number of layers but also the parameters of each layer. For the data at hand, we apply fivefold cross validation to get the optimal CNN architecture. The training templates are randomly partitioned into five equal-sized subsamples, where one subsample is retained as the validation data for testing the classifier and the remaining four subsamples are used as training data. We repeat the cross-validation process for five times to obtain our CNN classifier, which yields a classification accuracy of 92.39%, as shown in Fig. 5. The first convolutional layer has 20 filters with a filter size of 5×5, and the second convolutional layer has 20 filters with a filter size of 10×10. The pool size of both max-pooling layers is 2×2. The output sizes of the first and second fully connected layers are 10 and 2, respectively.

We then apply the resulting CNN classifier to classify the pixels in our experimental phase-contrast images through a sliding window manner. The centers of the clusters, which are classified as positive class, are selected as the position of cell nuclei.

### 2.4 Multiple Hypothesis Kalman Filtering

We combine backward Kalman filtering with MHT to associate and track the cell candidates within the vessels over time. Instead of starting with the image at the first time point, we initialize our tracks with the end-point confocal image, which provide the accurate cell locations and where all proliferation has occurred. Furthermore, we combine biological knowledge, such as vessel information and cell proliferation rate, to add constraints when estimating the probabilities in cell association.

We denote each individual cell state by $x_{k}={\left[x_{k},y_{k},{\overset{˙}{x}}_{k},{\overset{˙}{y}}_{k}\right]}^{T}$, where $x_{k}$ and $y_{k}$ are the estimated cell centroid positions in the x- and y-axes, respectively, and ${\overset{˙}{x}}_{k}$ and ${\dot{y}}_{k}$ are their corresponding velocities. The system covariance at time k is denoted by $P_{k}$. The observation state is the cell position in x- and y-axes obtained in cell detection process, denoted by $z_{k}={\left[x_{k},y_{k}\right]}^{T}$.

The backward Kalman filter consists of two steps: backward prediction and update. The prediction equations for the prior state estimate x^k−1|k and corresponding system covariance estimate $P_{k-1|k}$ are

x^k−1|k=Fx^k|k, (2)

$$P_{k-1|k}={\mathrm{FP}}_{k|k}F^{T}+Q,$$ (3)

and the update equations for the posterior cell estimate and covariance estimate are

x^k−1|k−1=x^k−1|k+Kk−1y˜k−1, (4)

$$P_{k-1|k-1}=(I-K_{k-1}H)P_{k-1|k},$$ (5)

where F is the state transition model, Q is the covariance matrix of the process noise, H is the observation model, which maps the state space into the observation space, R is the covariance of the observation noise, ${\overset{˜}{y}}_{k-1}$ is the innovation or the residual error between the predicted and observed estimate, $S_{k-1}$ is the innovation covariance, and $K_{k-1}$ is the optimal Kalman gain matrix, yielding the minimum mean square error. ${\overset{˜}{y}}_{k-1}$, $S_{k-1}$, and $K_{k-1}$ are calculated as follows:

y˜k−1=zk−1−Hx^k−1|k, (6)

$$S_{k-1}={\mathrm{HP}}_{k-1|k}H^{T}+R,$$ (7)

$$K_{k-1}=P_{k-1|k}H^{T}{\left({\mathrm{HP}}_{k-1|k}H^{T}+R\right)}^{-1}.$$ (8)

We assume a constant velocity model; hence, we have

$$F=[\begin{array}{cccc} 1 & 0 & \Delta t & 0\\ 0 & 1 & 0 & \Delta t\\ 0 & 0 & 1 & 0\\ 0 & 0 & 0 & 1 \end{array}]\;\text{and}\;H=[\begin{array}{cccc} 1 & 0 & 0 & 0\\ 0 & 1 & 0 & 0 \end{array}],$$ (9)

where Δt=−1 day.

**Fig. 7:**
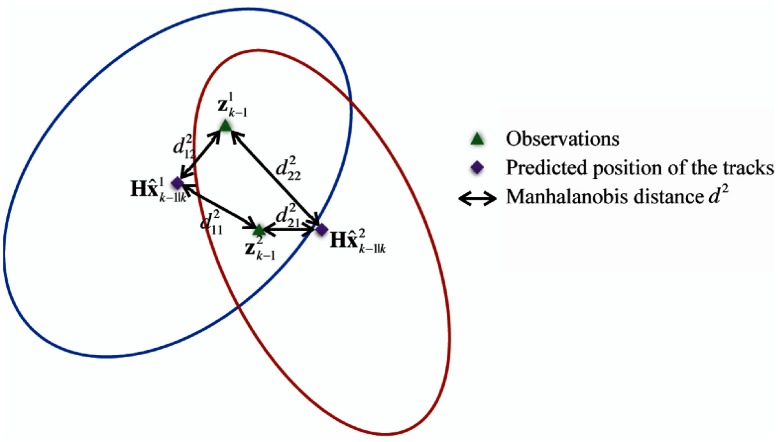
Backward Kalman filter with validation gating in data association: blue and red ellipses are the validation gate for tracks 1 and 2, respectively. Track 1 is associated with observation 1 and track 2 is associated with observation 2 in this plot.

Backward Kalman filter with validation gating is a popular approach to update the cell state during cell association. In each image, we extract multiple cells, which will be updated to multiple tracks. To associate an observation to a particular track, we employ a validation gate as an association threshold of whether to accept or reject an observation-to-target association. Observations that fall within the validation gate will be updated. As shown in Fig. 7, the blue and red ellipse are the validation gate for cell 1 and cell 2, respectively. Specifically, we compute the Mahalanobis distance between each observation to each state estimate as

$$d_{ij}^{2}={\overset{˜}{y}}_{ij,k-1}^{T}S_{ij,k-1}^{-1}{\overset{˜}{y}}_{ij,k-1},$$ (10)

where ${\overset{˜}{y}}_{ij,k-1}$ is the innovation between the backpredicted track i and the observation j and $S_{ij,k-1}$ is the corresponding innovation covariance. The observation, whose Mahalanobis distance with a track is smaller than a chi-squared threshold (validation gate), is more likely belonging to this track. If there are no observations within this gate, no observation updates to this track will occur. When there are multiple observations in a validation gate, the estimate is updated with only the best-matched observation (smallest Mahalanobis distance). For instance, observation 1 is associated with track 1 and observation 2 is associated with track 2 in Fig. 7.

**Fig. 8:**
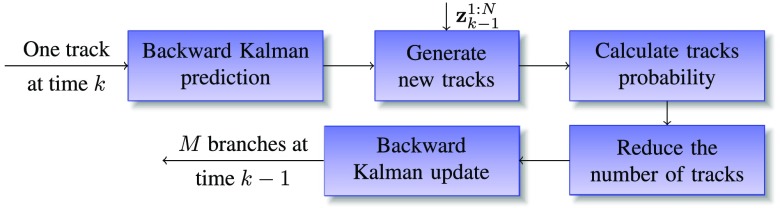
Flowchart of multiple hypothesis Kalman filtering. Cell state, system covariance, and probabilities of all the tracks are recursively updated over time to form multiple hypotheses. The cell associations are obtained by selecting the most probable hypotheses.

The alternative way for multiple observations problem is to apply MHT, where the association of observations can be delayed till sufficient information is available. The flowchart in Fig. 8 shows a diagram of the proposed cell tracking system.

**Fig. 9:**
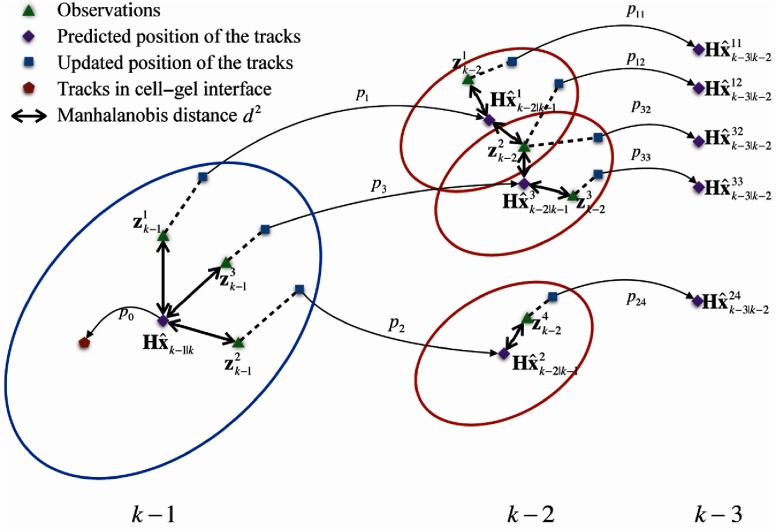
MHT for one cell in two successive time steps.

At the end point, which is the initial time point of the backward Kalman filter, we create one track for each cell. Each cell state and covariance estimate at time k are backpropagated to time k−1 using Eqs. (2) and (3). If there are $N_{k-1}$ observations which fall within the validation gate, MHT updates all the observations to the same track by generating $N_{k-1}$ new branches to the track. Figure 9 provides an example of the MHT process for one cell in two successive time steps. Within the blue ellipse, the purple diamond represents the predicted position of one track at time k−1. The green triangles indicate the observations that may be associated with this track with probability $p_{1}$, $p_{2}$, and $p_{3}$, respectively. The red pentagon depicts the hypothesis (with probability $p_{0}$) that the track is coming from the cell–gel interface. All the possible hypotheses in step k−1 are maintained and considered independently to generate new hypotheses in the next time step, as indicated by the red ellipses in Fig. 9. Since the number of branches for each track increases exponentially, to keep the computation under control, we maintain M branches for each time by including biological knowledge and pruning the tracks with low probability.

Let us consider cell i at time k. This cell might be due to the migration or proliferation of an existing cell labeled as j at time k−1. It is also possible that this cell is in the cell–gel interface or out of focus (not in the focal plane) at time k−1. The corresponding probabilities are calculated in different ways.

We employ the Mahalanobis distance to calculate the probability that track i stems from the migration or proliferation of an existing cell j. If the distance is smaller than the threshold gate value, the probability that the track i is associated with observation j is calculated as

$$p_{i}(z_{k-1}^{j})\propto {\left|2\pi S_{ij,k-1}\right|}^{-\frac{1}{2}}e^{-\frac{1}{2}d_{ij}^{2}}.$$ (11)

We assume that the cells nearer to the cell–gel interface (larger y-position) are more likely to be new tracks. The probability that track i is due to migration from the cell–gel interface is computed as

$$p_{i}(M)\propto \frac{1}{{\left(b_{1}-y_{k-1|k}\right)}^{b_{2}}},$$ (12)

where $b_{1}=1700$ and $b_{2}=2.47$ are the empirical parameters tuned from our experimental data and $y_{k-1|k}$ is the cell position in y-direction from backward Kalman prediction. The probabilities for track i estimated by Eqs. (11) and (12) are then normalized for this time step.

We include biological knowledge to reduce the number of the new tracks in each step. Since the doubling time of our ECs is 15 to 48 h, we assume that one cell can at most split once within one day. In other words, we allow at most two cells at time k to be associated with one same observation at time k−1 during merging. In our angiogenic vessels, cells cannot overtake each other due to the existence of tight cell junctions, so we eliminate the tracks where overtaking happens during association. Meanwhile, we discard the tracks with low probability to keep the number of tracks generation computations under control.

As mentioned, we keep M branches and obtain their corresponding cell state estimates and system covariance estimates with backward Kalman update for each time step. Typically, M=4 is selected since the remaining branches have negligible probabilities. Each of these new tracks is considered independently and used to generate new predictions for next time step. We repeat this tracking process until k=1 and obtain $N_{0}M^{t-1}$ hypotheses in total, where $N_{0}$ is the number of the cells obtained from the end-point confocal images and t is the total time steps. Last, the tracks are selected as the most probable hypotheses.

## 3 Results and Discussion

We applied the proposed automated multicell tracking system to the experimental time-lapse phase-contrast images with the aim of tracking the nuclei of the ECs within the angiogenic vessels.

### 3.1 Image Registration Results

**Fig. 10:**
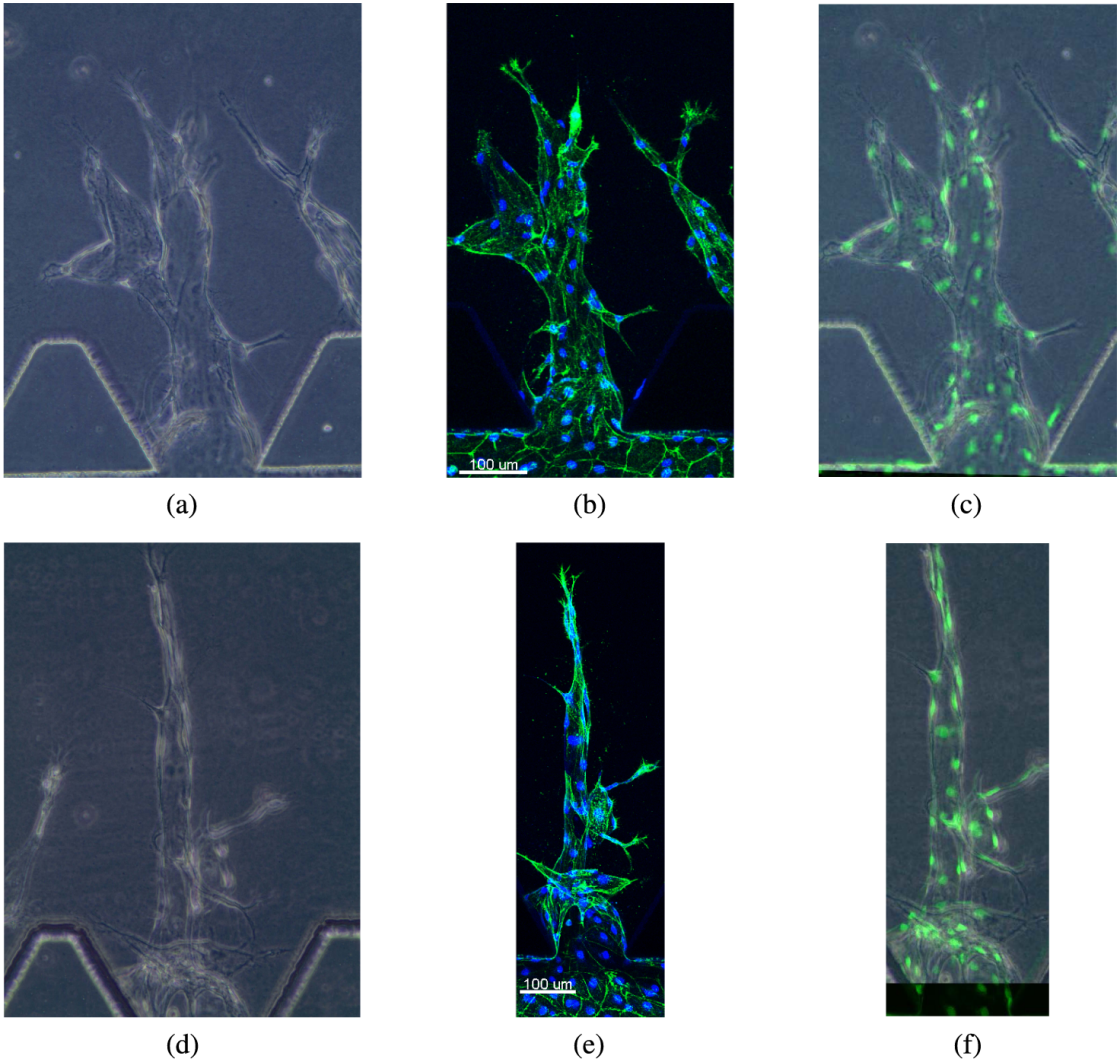
Illustration of image registration results: (a) and (d) are the end-point phase-contrast images, (b) and (e) are their corresponding confocal images, and (c) and (f) show the coregistered confocal and phase-contrast images.

With our proposed registration algorithm, we aligned all the phase-contrast image sequences and their corresponding end-point confocal images. We overlay the end-point phase-contrast images and their corresponding end-point confocal images to illustrate the registration results (see Fig. 10).

Figures 10(a) and 10(d) are the original end-point experimental images obtained from phase-contrast microscopy. Figures 10(b) and 10(e) are their corresponding confocal images. Here, the blue blobs are the cell nuclei stained by Hoechst, and the green structures are the actin stained by Phalloidin. We can see that the size and position of the vessels are different in the phase-contrast images and confocal images. The aligned phase-contrast and confocal images are shown in Figs. 10(c) and 10(f), where the resized phase-contrast images are shown in the background. We marked the cell nuclei stained in the confocal images as green blobs for clearer identification. The angiogenic structures are well-aligned in Fig. 10, suggesting our image registration algorithm is accurate.

### 3.2 Cell Detection Results

**Fig. 11:**
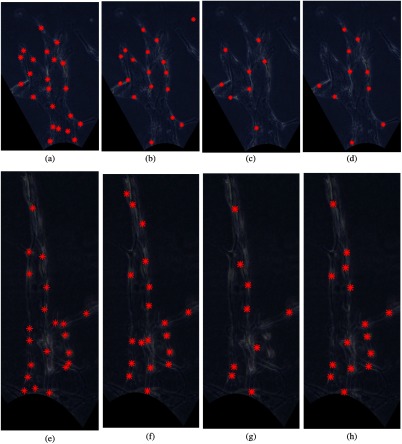
Cell detection results of examples in Fig. 10 from different approaches: (a) and (e) from CNN classifier, (b) and (f) from PLSR,^47^ (c) and (g) from PCA in conjunction with SVM, and (d) and (h) from an SVM classifier. Red stars (*) indicate the locations of the detected cell nuclei.

We consider the same examples as in Sec. 3.1 to illustrate our cell detection results. Figures 11(a) and 11(e) show the results for the proposed CNN-based cell detection algorithm. Figures 11(b) and 11(f) show the results from partial least square regression (PLSR) approach with 30 PLS components as explained in Ref. 47. Figures 11(c) and 11(g) show the cell detection results for principal component analysis (PCA) in combination with SVM classification. We also trained an SVM classifier based on the original high-dimensional template data to distinguish the cells from the background in our experimental images, for comparison. The results are shown in Figs. 11(d) and 11(h). The red stars (*) label the location of the detected cell nuclei for all these images.

To evaluate the performance of these approaches quantitatively, precision and recall measures are calculated. Precision is the fraction of detected cells that are actual cells, and recall is the fraction of the actual cells that are detected

$$\text{Precision}=\frac{\mathrm{TP}}{\mathrm{TP}+\mathrm{FP}}\;\text{and}\;\text{Recall}=\frac{\mathrm{TP}}{\mathrm{TP}+\mathrm{FN}},$$ (13)

where TP, FP, and FN are defined in the 2×2 confusion matrix in Table 1.^48^ This confusion matrix describes the four possible outcomes of a given binary classifier and a set of instances.

**Table 1:** Confusion matrix for a binary classifier.^48^

	Actual positive	Actual negative
Assigned positive	True positive (TP)	False positive (FP)
Assigned negative	False negative (FN)	True negative (TN)

To assess the different cell detection approaches, we also compute F-score $F_{1}$, defined as the harmonic mean of precision and recall

$$F_{1}=2\cdot \frac{\text{Precision}\cdot \text{Recall}}{\text{Precision}+\text{Recall}}.$$ (14)

To validate the cell detection results, we use the end-point phase-contrast images, matching the estimated cell locations with their corresponding confocal images. We analyzed 18 end-point phase-contrast images in this paper. Since the phase-contrast image is a 2-D slice and the confocal image is in 3-D with multiple slices, we choose the ground truth in two ways. For the first case, the actual positives and negatives are extracted from all the slices of confocal images. However, since our phase-contrast image is in 2-D, we use only one slice of the confocal image to provide the ground truth for the second case. We choose the slice that has a similar focal plane as the 2-D phase-contrast image. The TP, FP, and FN values for the four approaches are provided in Table 2. Their corresponding precision, recall, and $F_{1}$ values are listed in Table 3.

**Table 2:** TP, FP, and FN values for different cell detection approaches.

	Case 1: all slices			Case 2: one slice		
Detection approaches	TP	FP	FN	TP	FP	FN
CNN	228	33	38	228	33	22
PLSR	217	38	49	217	38	33
PCA and SVM	107	26	159	107	26	143
SVM	124	23	142	124	23	126

**Table 3:** Classification performance for different cell detection approaches.

	Case 1: all slices			Case 2: one slice		
Approaches	Precision (%)	Recall (%)	$F_{1}$	Precision (%)	Recall (%)	$F_{1}$
CNN	87.4	85.7	0.87	87.4	91.2	0.89
PLSR	85.1	81.6	0.83	85.1	86.8	0.86
PCA and SVM	80.5	40.2	0.54	80.5	42.8	0.56
SVM	84.4	46.6	0.60	84.4	49.6	0.62

For case 1, all the precision values are above 80%, suggesting that most of the detected cells are actual cells. The recall for CNN reaches 85.7%, which is higher than for the other three approaches, indicating that CNN detects most of the actual cells.

The recall of CNN is limited to about 85.7%, which can be explained as follows. The height of our device is around 120  μm and there are cells migrating throughout this range. We only acquire the 2-D image of the angiogenic vessels in focus. Some cells, which are external to the vessels, are out of focus and almost invisible in the phase-contrast images. These cells are not detected by our algorithms. However, these out-of-focus cells are observed in the 3-D confocal images. This scenario can be seen in Fig. 10.

For case 2, the recall for all the four approaches increases as shown in Table 3. Particularly, the recall for the proposed cell detection algorithm (CNN) increases to 91.2%. From the values of F-score in Table 3, which balances recall and precision, we can also conclude that CNN is a preferable algorithm to detect the ECs within the 3-D angiogenic vessels from our experimental phase-contrast images. We hypothesize that the features extracted from CNN are more suitable to represent the cell templates.

### 3.3 Tracking Results

Since our cell association approach is detection based, we use the cell detection results from the proposed approach based on CNN as the cell locations during cell association.

**Fig. 12:**
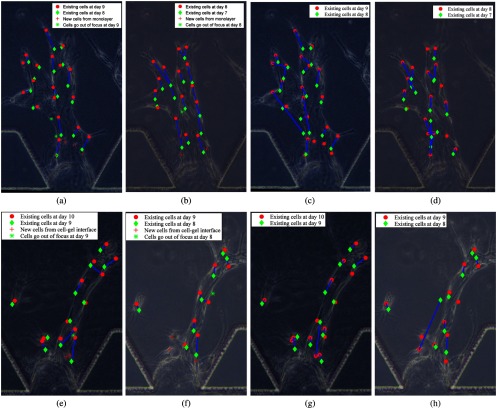
Cell tracking examples: (a)–(d) for example 1 and (e)–(h) for example 2. (a), (b) and (e), (f): backward Kalman filter with MHT, (c), (d) and (g), (h): backward Kalman filter with validation gating. Each image shows the outcomes of the tracking algorithms for two successive frames, where the phase-contrast image is shown in the background.

We provide two examples of the association results in Fig. 12. The results for the proposed tracking system are shown in Figs. 12(a), 12(b), 12(e), and 12(f). We also apply backward Kalman filter with validation gating for cell association [see Figs. 12(c), 12(d), 12(g), and 12(h)]. Each image presents the tracking results for two successive frames and the background is the experimental phase-contrast image. We can see that the proposed multicell tracking approach produces better tracking results than backward Kalman filter with validation gating.

We applied the proposed system to track cells in five sprouting slots, where each slot has four to five frames (one frame per day). We estimated the association accuracies (the percentage of the correct associations in the total associations) for the proposed approach, the nearest-neighborhood association, the backward Kalman filter with validation gating, and the Hungarian method^32^ (see Table 4). We excluded in this comparison methods in the literature that require more information about the cells than merely the cell locations, since such information is unavailable for our data. Since these methods cannot be applied to our data, we did not assess them. We obtained the ground truth through manual association, which is the traditional method of analyzing the cell migratory behaviors.

**Table 4:** Accuracy (percentage of correct associations) for different cell association approaches. I, nearest-neighborhood association; II, Kalman filter with validation gating; III, Hungarian method; and IV, Kalman filter with MHT.

	Correct associations	Total associations	Association accuracy (%)
I	53	198	26.7
II	119	198	60.1
III	105	198	53.0
IV	171	198	86.4

Since the sampling interval is one day, cells migrate over a relatively long distance between frames. Using nearest-neighborhood association, the new tracks at time k are typically associated wrongly to cells that are near to the cell–gel interface at time k−1. In the backward Kalman filter with validation gating approach, we predict the track backward each time step via a constant velocity motion model. In this model, we assume that the track velocity is a constant during one time step, and we update it at every time step. This approach yields more reliable association results, improving the association accuracy from 26.7% to 60.1%. In the Hungarian method, we first predict the new positions of the cells at time k using a constant velocity model and then estimate the Euclidean distances of the predicted cells at time k and the cells at time k−1, to find the associations with minimal cost. However, cell migration and proliferation are not considered since it only allows for one-to-one matching. Thus, only 53.0% association accuracy is obtained. In our approach, we combine MHT with backward Kalman filter, which delays the decision making, and determine the associations once sufficient information is available. Moreover, biological knowledge is incorporated when considering the new cells from the cell–gel interface and reducing the number of new tracks. As a result, we can track not only the migration and proliferation of the existing cells but also the new cells from cell–gel interface and out-of-focus plane (see Fig. 12). This explains why the association accuracy increases to 86.4% for our approach. Compared with the association accuracy (85.9%) based on PLSR in Ref. 47, we can also conclude that the increment in cell detection accuracy due to the CNN improves the association accuracy.

**Fig. 13:**
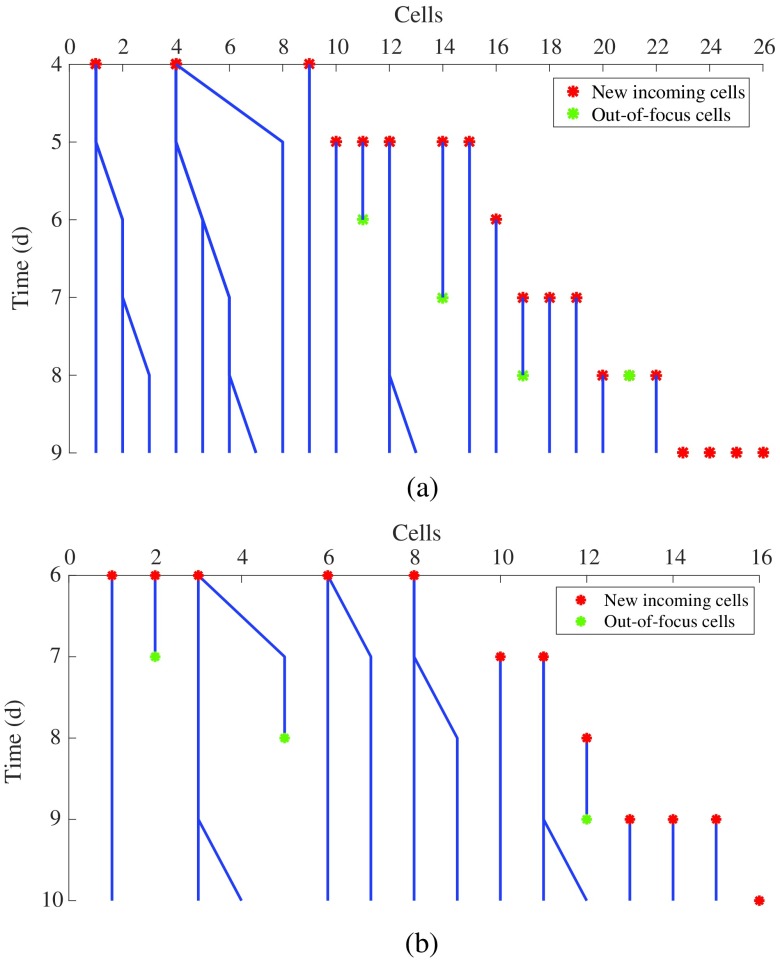
Cell lineage plot: (a) for example 1 and (b) for example 2 in Fig. 12. The blue lines represent the cells that migrate within the 3-D gel; the red stars represent the incoming cells from the cell–gel interface, whereas the green stars indicate the cells migrating out of focus.

Cell trajectory information can be obtained from the tracking results. Moreover, the system can automatically generate cell lineage plots (see Fig. 13), showing the history of the cell proliferation and cell migration into the gel, with timestamps of when it is in focus and out of focus. Such cell lineage plot provides crucial information (e.g., the number of cells, division time, growth fraction, etc.) to biologists who are interested in studying cell migration behaviors under different conditions.

The cell lineage plots in Figs. 13(a) and 13(b) correspond to the two examples in Fig. 12. The blue lines indicate that cells migrate within the 3-D gel. The branch points of these blue lines represent cell proliferation. The red stars illustrate the incoming cells from the cell–gel interface. The green stars indicate that cells migrate out of focus. The cell lineage plot illustrates the history of cell migration and proliferation in a compact and effective manner.

By comparing the cell lineage plots obtained under different experimental conditions, biologists can easily figure out the influences of the chemical factors on cell division and cell migration. For instance, Fig. 13(a) is from angiogenic experiment under positive sphingosine-1-phosphate (S1P) gradient, and Fig. 13(b) is from angiogenic experiment without S1P.^9^ By comparing these two figures, one can conclude that positive S1P gradient induces more recruitment of cells from the monolayer and stimulates cell proliferation. The cell lineage plots provide quantitative information about the influence of S1P on angiogenesis. Such quantitative data are an important step toward better understanding and new theories of angiogenesis; hence, the proposed tools probably will enable biologists to make new discoveries concerning angiogenesis. These data can also be used to develop mathematical models to predict the cell migration under different conditions, leading ultimately to a better understanding cell migration in angiogenesis.

## 4 Conclusion

We presented an automated image analysis system to track the migrating ECs within 3-D angiogenic vessels cultured in MFDs by combining end-point confocal and time-lapse phase-contrast images. This system consists of image registration, vessel segmentation, cell detection, and multiple hypothesis Kalman filtering to track cell proliferation and migration (in/out of focus) in 3-D angiogenic vessels. We incorporate biological knowledge, such as vessel information and experimental device properties, to add constraints and to estimate the track probability during cell association. Our proposed cell detection algorithm based on CNN yields 87.4% precision and 91.2% recall for cell candidates detection, which helps the biologists to recognize a large number of cells automatically with high accuracy. The association accuracy reaches 86.4% for the proposed multiple hypothesis Kalman filtering approach when associating the detected cell candidates. In the future, we will apply deep residual learning to improve cell detection accuracy. We will also explore other possible features of cells and combine the detection likelihood of each cell with the Mahalanobis distance to improve the association accuracy. From the tracking results (see Fig. 12), we are able to obtain information about the cell trajectory and create a cell lineage plot (see Fig. 13) to visualize the history of the cell migration and proliferation in a compact and effective manner. By comparing with the identified biomarkers in the end-point confocal images, biologists can explore links between the chemical and physical characteristics of a cell. With this information about the cell history, mathematical models can be developed to predict the cell migration under different cell conditions, leading ultimately to a better understanding of the biological processes in cells.
